# Ventricular coupling in single ventricle patients: a MRI study of cardiac biomechanics

**DOI:** 10.1186/1532-429X-18-S1-P163

**Published:** 2016-01-27

**Authors:** Ramkumar Krishnamurthy, Cory V Noel, wei Pan, Jeffrey Jacot, Regina Lantin-Hermoso, Rajesh Krishnamurthy

**Affiliations:** 1 grid.416975.80000000122002638Radiology, Texas Children's Hospital, Houston, TX USA; 2 grid.39382.33000000012160926XPediatric Cardiology, Baylor College of Medicine, Houston, TX USA; 3 0000000419368278Bioengineering, Rice University, Houston, TX USA

## Background

Ventricular dysfunction in patients with a single right ventricle (SRV) or a single left ventricle (SLV) is a known risk factor for morbidity and mortality. In normal hearts, LV and RV augment each other, while this is not possible in single ventricle (SV) patients. Ventricular-ventricular relationship in SV patients remain poorly understood, with only a few studies performed [1-3]. Our earlier results show a decreased peak circumferential (ε_cc_) and longitudinal (ε_L_) strain in SV patients when compared to a normal population [4]. However, an increase in longitudinal strain is noted in regions of decreased circumferential strain.

The purpose of this study is to understand the ventricular-ventricular interactions in systemic ventricles coupled and uncoupled to a dysfunctional ventricle.

## Methods

We performed a prospective, IRB approved study of 24 subjects ( 9 normal age: 11.8 +/- 3; 8 SRV age: 11.4 +/- 2.3; 7 SLV age: 12.7 +/- 4.2 years). SLV and SRV patients were asymptomatic at time of imaging and were post total cavo pulmonary connection (TCPC).

Acquisition Protocol:

Strain information was acquired at three short axis slices at basal (coupled to dysfunctional ventricle), and apical (uncoupled) locations in all 18 subjects in a 1.5T MRI scanner (Philips Acheiva) using: a) Complementary Spatial Modulation of Magnetization (CSPAMM)^4^ images: ε_cc_; and b) Fast-Strain Encoded (fSENC)^5^ images: ε_L_.

Data Analysis:

ε_cc_ and ε_L_ across all cardiac phases were calculated from SAX slices using Diagnosoft^TM^. Global, free-wall and septal strain were calculated at both locations and ventricular coupling index (VCI) is calculated as (ε_cc_ * ε_L_ /100).

## Results

**Figure 1:**
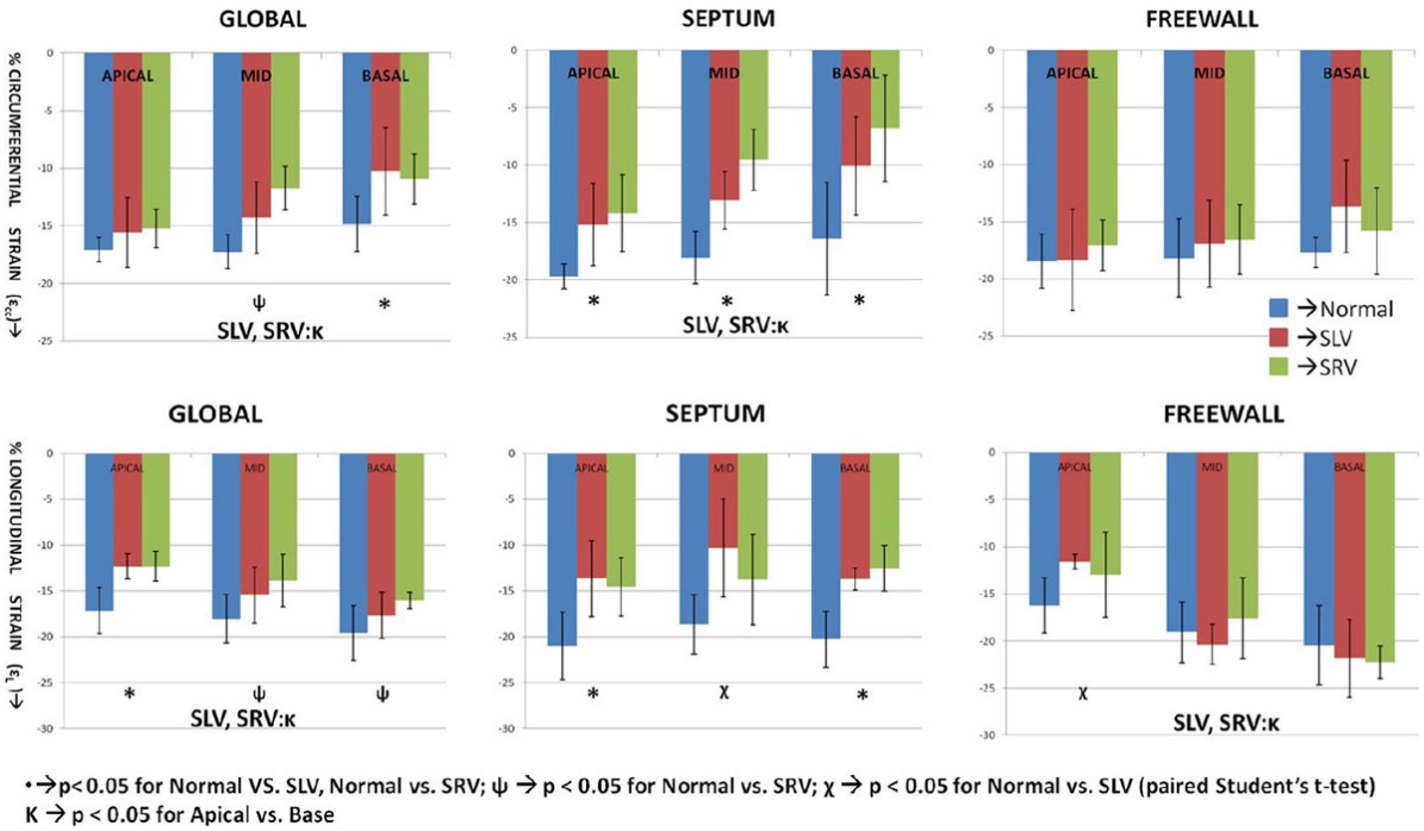
**Bar plots showing the longitudinal (ε**_**L**_**) and circumferntial (ε**_**CC**_**) strain in a pediatric single venricle population**. We demonstrate a significant reduction in both ε_CC_ and ε_L_. The septum is the most affected with negligible differences observed in free wall. Also, there is a signficant difference observed from apex to base globally for both single systemic ventricle patients, while the free wall ε_L_ shows a significant increase.

1. Strain values of SLV and SRV subjects demonstrate significant differences compared to normal subjects. (Figure 1)

2) Strain at the septal location is significantly reduced in single ventricle patients while the freewall strain is relatively normal.
3) Circumferential strain of the SV progressively reduces from the apex to the base, while the longitudinal strain increases.
4) VCI is significantly reduced at the basal septum - pointing to the deleterious effect of the ventricular coupling between the systemic ventricle and the dysfunctional ventricle. Myocardial fiber arrangement and the hypoplastic chamber likely affect the regional differences demonstrated in this study (Figure 2).

**Figure:**
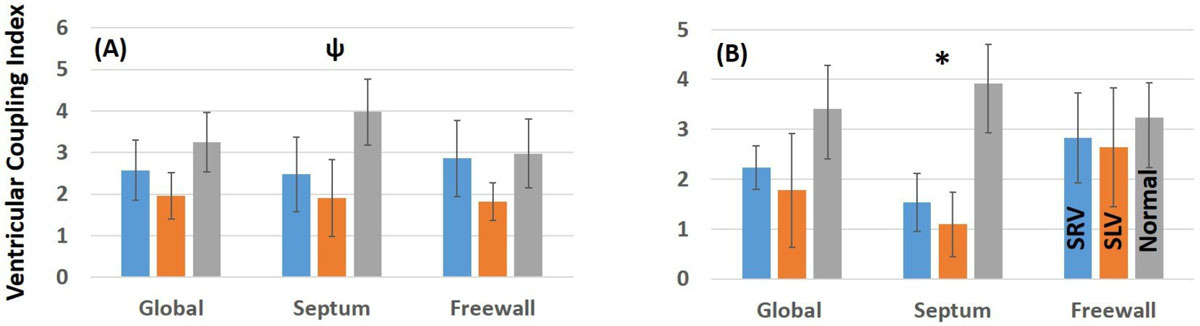
Figure 2

## Conclusions

Longitudinal strain is increased in regions where the circumferential strain is decreased in SV patients. However at the basal septum, where the dysfunctional ventricle is attached to the septum, it appears that the systemic ventricle mechanics is affected by deleterious ventricular coupling. Further studies are needed to understand differences between SRV and SLV cardiac biomechanics.
